# A new systematization of histological analysis for the diagnosis of Hirschsprung's disease

**DOI:** 10.1016/j.clinsp.2023.100198

**Published:** 2023-04-18

**Authors:** Suellen Serafini, Maria Mercês Santos, Ana Cristina Aoun Tannuri, Celso Di Loreto, Josiane de Oliveira Gonçalves, Uenis Tannuri

**Affiliations:** a Pediatric Surgery Division, Pediatric Liver Transplantation Unit and Laboratory of Research in Pediatric Surgery (LIM 30), Hospital das Clinicas, Faculdade de Medicina, Universidade de São Paulo (HCFMUSP), São Paulo, SP, Brazil; b Department of Pathology, Hospital Alemão Oswaldo Cruz, São Paulo, SP, Brazil

**Keywords:** Hirschsprung's Disease, Rectal biopsy, Ganglion cells, Submucosa

## Abstract

**Background:**

Hirschsprung's Disease (HD) is characterized by intestinal sub-occlusion and the absence of enteric ganglion cells. A rectal biopsy examination is performed to confirm the diagnosis. In a recent study, we demonstrated that the analysis of 60 sections of rectal mucosa and submucosa stained by H&E may ensure a 90% diagnostic accuracy. Although the need to analyze so many sections makes the process of reading the slides more time-consuming, this encouraged us to study their distribution in the healthy rectal submucosa, to simplify the diagnosis.

**Objectives:**

To develop a method that facilitates HD diagnosis by studying the distribution of ganglion cells in the submucosal plexus.

**Methods:**

Using the calretinin technique, we studied the distribution of plexuses in 60 fragments of rectal submucosa from 19 cadavers. After the study, the reading method created was used for diagnosis in 47 cases of suspected HD, using H&E staining. The accuracy was verified by comparing the results obtained with H&E to those obtained with the acetylcholinesterase technique, the golden standard in our laboratory.

**Results:**

The study of submucosal plexus distribution showed that just by examining the submucosal region every 20 µm, approximately, it is possible to locate a ganglionic plexus, and we have already been able to diagnose HD with 93% accuracy.

**Conclusion:**

The study of ganglion cell distribution enabled the creation of a simplified method for reading the slides. The method applied achieved good accuracy and it can be used as an alternative method in HD diagnosis.

## Highlights

• Hirschsprung's disease is a congenital disorder characterized by intestinal sub-occlusion and the absence of neurons in the enteric nervous system, with involvement of varying segments of the large intestine.
• The treatment is only surgical, for this reason the diagnosis must have a good accuracy.
• This research demonstrates a simplified method to perform the diagnosis with 93% of accuracy.
• The use of this method brings benefits to the patient, the pathologist, and the service.

## Background

Hirschsprung's Disease (HD), also known as congenital megacolon, is characterized by intestinal sub-occlusion with an underlying pathology marked by the absence of neurons in the enteric nervous system, affecting different segments of the large intestine.^1^^,^^2^

Although the disease was first described in 1886 by Harald Hirschsprung, it still poses a challenge, both in terms of its etiopathogenesis and its genetic aspects.^2^, ^3^, ^4^ After the pathophysiological characterization of HD in 1948 by Whitehouse and Kernohan,^1^ rectal biopsy became the method of choice for confirming the diagnosis. However, there is still controversy about the best technique for preparing the biopsy material. In 1955, Swenson demonstrated the possibility of performing the diagnosis with 98% accuracy in fragments of the total rectal wall using H&E staining, since the identification of neurons in the myenteric plexuses rules out the diagnosis.^5^ Nonetheless, this biopsy requires general anesthesia, which makes the procedure more invasive and more difficult to perform, especially in newborns.^6^ Five years later, Bodian^7^ showed that HD diagnosis could be less invasive in smaller fragments, containing only mucosa and submucosa, utilizing H&E staining. Unfortunately, this method is not universally well accepted due to the dispersed distribution of the ganglion plexuses in the submucosal layer, which makes the diagnosis more difficult.^8^

Therefore, when Meier-Ruge^9^ described the histochemical technique for AChE activity in 1972, it became the method of choice for HD diagnosis in many centers worldwide. In the case of HD, this histochemical method shows the presence of fibrils and hypertrophied nerve trunks.^10^^,^^11^ In addition, the technique has the advantage of being specific and it requires only a small fragment of mucosa and submucosa.^3^^,^^12^ In our Service, this method was elected as the gold standard for HD diagnosis. In 2008, Santos et al.^13^ demonstrated that AChE-stained biopsies provide 93.5% accuracy, by analyzing material from 297 children who underwent rectal biopsies. Despite being a specific and accurate diagnostic method, it requires specific reagents, a cryostat, and trained staff to carry it out. Finally, the rectal material biopsy may easily undergo autolysis if not immediately processed.^10^^,^^13^

In the last 30 years, advances in immunohistochemical techniques have made the utilization of calretinin markers feasible for HD diagnosis. Calretinin is a calcium-binding protein found in ganglion cells and thin fibrils in the colon mucosa lamina propria of patients with normal gut innervation. This method requires only a small fragment of mucosa and submucosa.^12^^,^^14^^,^^15^ Currently, we may affirm that the investigation of AChE activity and the immunohistochemical labeling of calretinin are the most widely used methods for HD diagnosis.

The major obstacle to the routine utilization of AChE activity and immunohistochemical labeling of calretinin for HD diagnosis is that it is not available in all medical centers. So, new studies have emerged aiming to simplify HD diagnosis, revisiting the use of H&E staining in rectal mucosa and submucosa fragments.^16^, ^17^, ^18^, ^19^ The detection of ganglion cells in the submucosal plexuses by utilizing H&E staining, as simple as the method might be, still creates a lot of controversies. Several authors cite the difficulty in identifying these cells, since they are diffusely distributed throughout the intestinal submucosa.^14^^,^^20^, ^21^, ^22^ We demonstrated, in a recently published study conducted at our laboratory, that it is possible to get an accurate HD diagnosis only with mucosal and submucosal fragments stained by H&E, as long as at least 60 sections of each fragment are analyzed.^18^ Using this method, we can accurately diagnose HD in a simpler and less invasive way, although the need to analyze so many sections makes the process of reading the slides more time-consuming, taking about 30 to 60 minutes.^20^ These findings aroused the interest in better studying the distribution of plexuses in the submucosal region of normal patients since there are no pre-existing studies concerning that matter. So, in the present study, we aimed to develop a model to systematize and facilitate the process of reading the slides. It is worth mentioning that this study will be of great value for pediatric surgery services that have no access to such specific diagnostic methods for HD, such as AChE and calretinin techniques.

## Methods

This study was approved by the Research Ethics Committee of the Institution (Process number 3.119.706). We also obtained a letter of assent from the director of the São Paulo city morgue for the use of cadavers, and a signed Informed Consent Form (ICF) from family members.

### Study of the distribution of submucosal plexuses in intestines with normal innervation

Nineteen fragments from the rectal wall of cadavers obtained from the city morgue (Serviço de Verificação de Óbitos de São Paulo ‒ SVOC-SP) between July 2019 and March 2020 were obtained with the consent of the respective family members. The donors were adults of varying ages and genders. The exclusion criteria for the collection of specimens were cadavers with < 12h postmortem, for quality reasons, as after this period tissues lose their integrity, and cadavers of people with Chagas disease, which may cause acquired megacolon in adults due to the loss of ganglion cells in the enteric plexuses.^23^

**Fig. 1:**
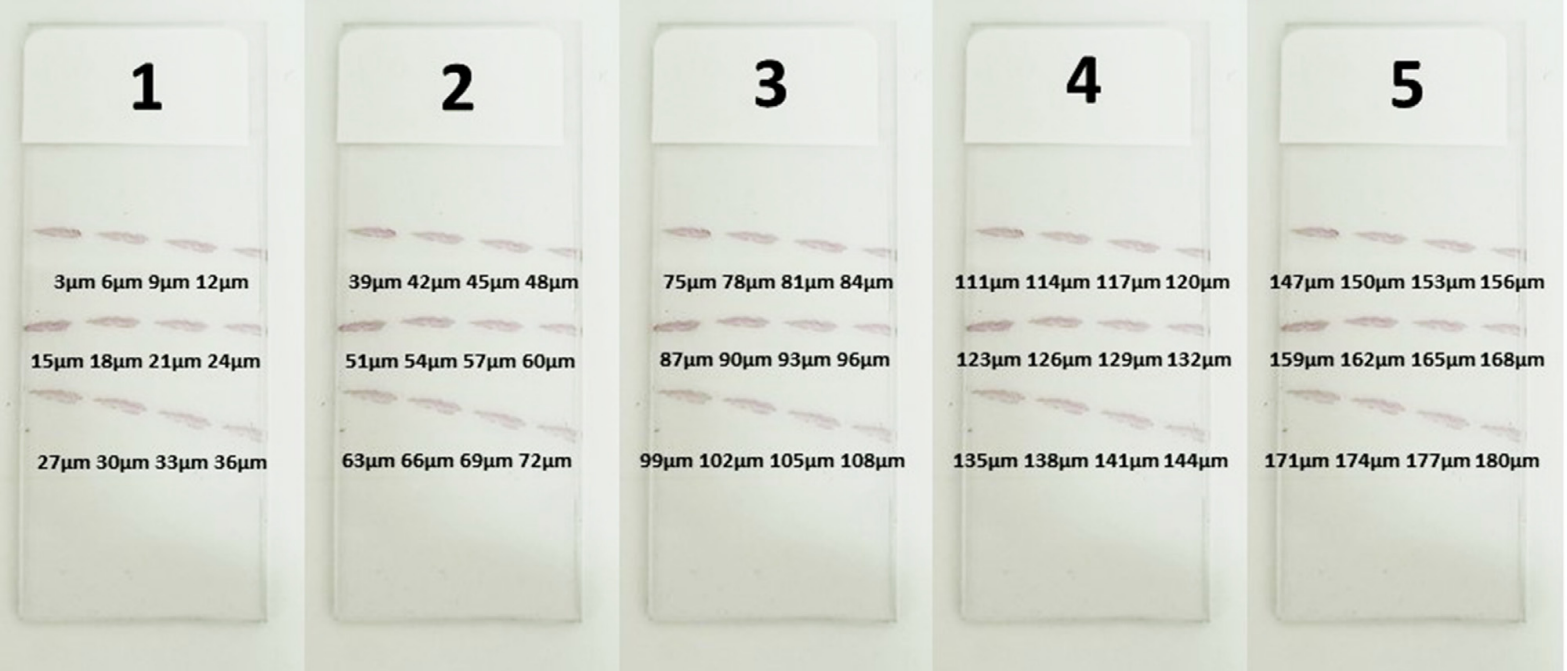
Slides layout demonstrating the position of the cuts.

**Fig. 2:**
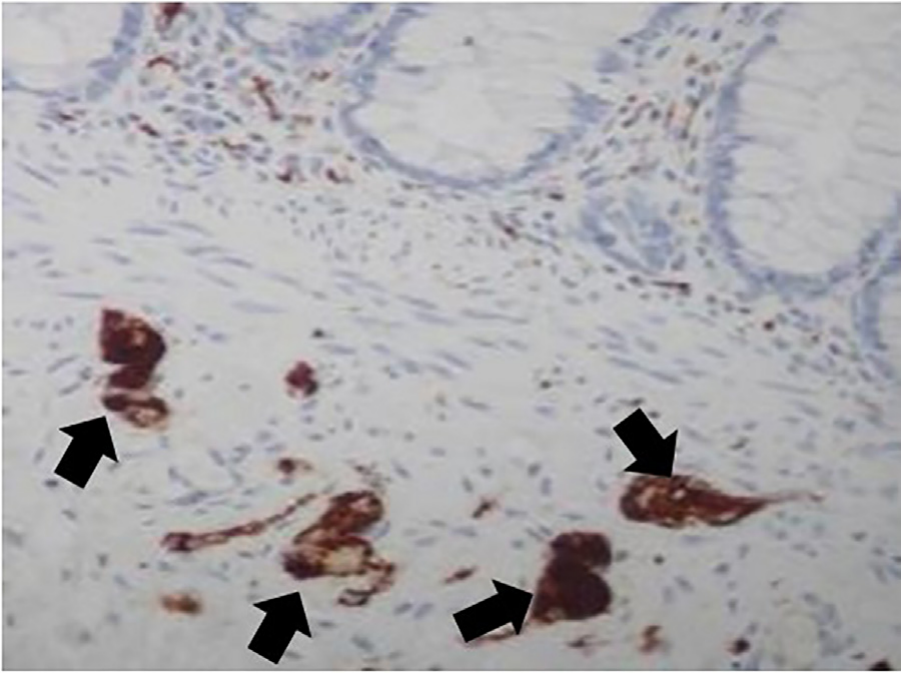
Calretinin labeling ganglion cells in submucosal plexus (400× magnification).

Initially, a block was collected from the terminal region of the bowel during a necropsy examination. Then, a small fragment was removed in our laboratory, simulating a rectal biopsy procedure. The collected specimens were placed in formalin solution for tissue preparation, in an automatic processor (Lupe PT05, Brazil). The samples were embedded in paraffin blocks following a perpendicular orientation. Using a microtome (Leica RM2255, Germany), sixty (60) 3 µm-thick sections were serially obtained from each fragment. We then applied the calretinin immunohistochemical technique using an antibody (Rabbit Anti-Human Calretinin Monoclonal Antibody – Clone SP13 – Abcam, USA). A total of 180 µm comprised of 60 serial 3 µm sections of each sample were analyzed to determine the location of the plexuses (Fig. 1, Fig. 2 and 2).

### Development of the model to systematize the analysis

After data collection, we systematized the material analysis by equidistance, checking the distance between the submucosal plexuses for each sample. We started the analysis by the extremities (3 µm and 180 µm) and inserted new measurements, always at the midpoint between the previous distances, until 100% accuracy was reached.

(a) Accuracy of the first and last 180-micron distance cut analyses.
(b) Accuracy of the first, 30th, and last 90-micron distance cut analyses.
(c) Accuracy of the first, 15th, 30th, 45th, and last 45-micron distance cut analyses.
(d) Accuracy of the first, 8th, 15th, 22nd, 30th, 37th, 45th, 52nd, and last 20-micron distance cut analyses.

### Applying the model to HD diagnosis

**Fig. 3:**
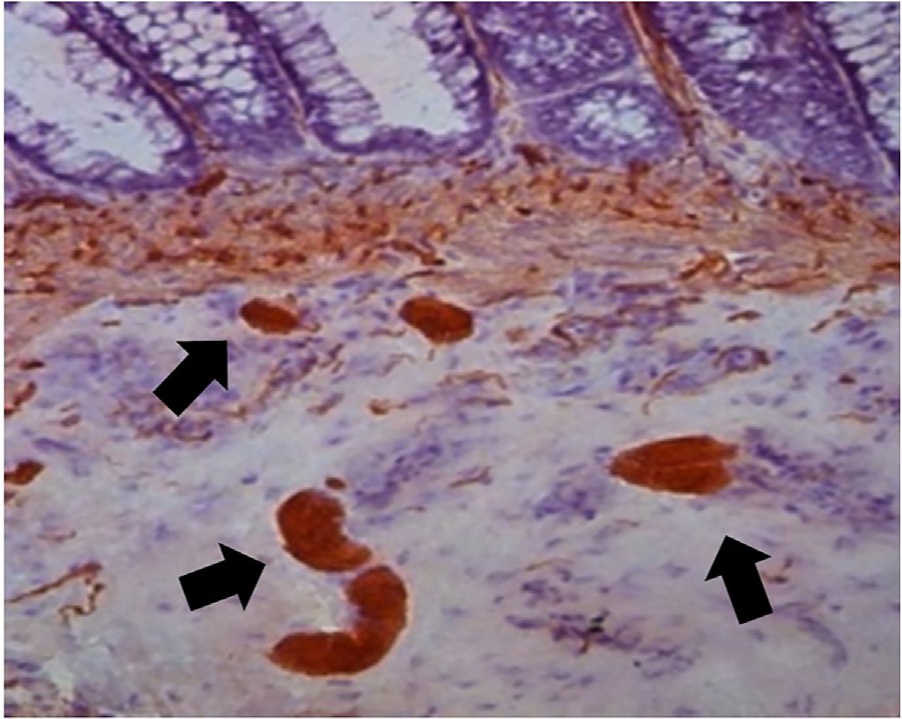
Acetylcholinesterase pattern for Hirschsprung's disease showing large quantities of cholinergic fibers (400× magnification).

**Fig. 4:**
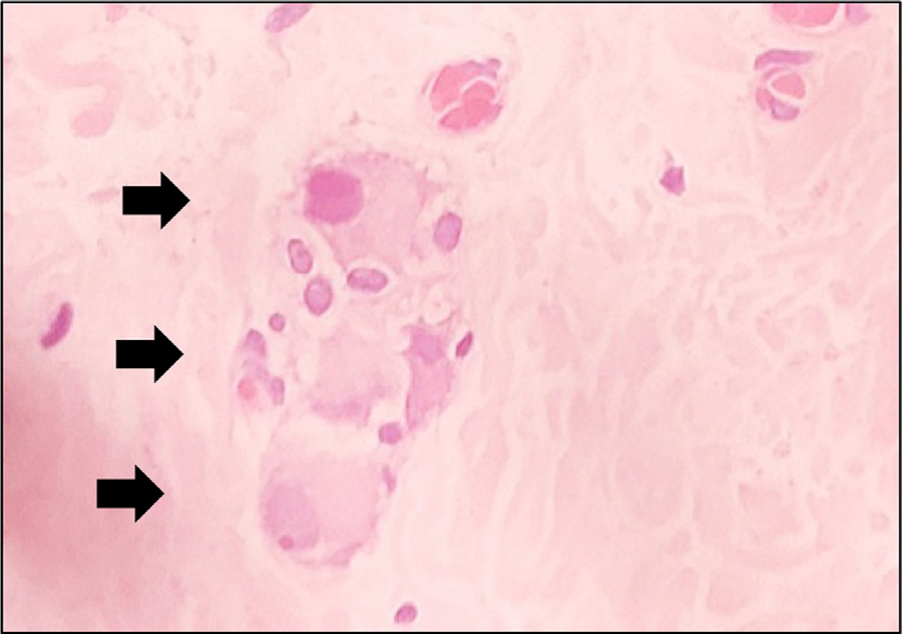
Submucosal plexus with 3 ganglion cells stained by Hematoxylin-Eosin (400× magnification).

This study examined 47 paraffin blocks prepared in our laboratory. These blocks contained biopsies to confirm HD diagnosis in patients treated at our institution, in the period ranging from 2016 to 2019. Two fragments of rectal mucosa and submucosa were collected from each patient. One fragment was frozen for AChE activity screening, and the other was embedded in paraffin and stored for further studies. The harvested fragment for AChE screening was processed by freezing and subsequently sectioned in a cryostat (Slee MEV, Germany). The AChE activity was demonstrated by the method of Karnovsky and Roots^11^^,^^24^ and counterstained by Carrazzi's hematoxylin (Fig. 3). The 47 specimens were also included perpendicularly and embedded in paraffin. They were sectioned with an electronic microtome (Leica RM2255, Germany). We utilized the same method for preparing the material, cutting 180 µm (60 cuts) from each sample, but we used H&E staining. The H&E-stained slides were examined using the proposed reading method to determine whether there were ganglion cells in the submucosal plexus; the observers had no information about the previous diagnosis made by AChE screening. The image below shows the submucosal plexus with three ganglion cells in evidence, 400× magnification (Fig. 4).

### Statistical analysis

Spearman's test was applied to verify if there was any correlation between donor age or postmortem time and the number of plexuses found in each sample. To analyze the matches between H&E staining and AChE technique, i.e., the golden standard, we used the Kappa and Chi-Square statistical model for sensitivity and specificity after the contingency tables were put together. R software version 4.0.2 was used to perform the statistical analyses.

## Results

### Study of ganglion cell distribution in samples with normal rectal innervation

**Fig. 5:**
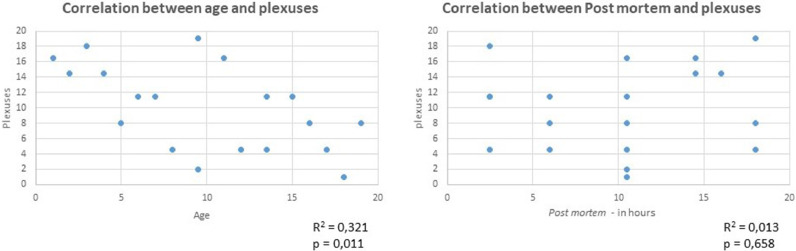
Graphic demonstrating the values of correlation between age and *postmortem* × number of plexuses.

Among the 19 cadaver donors who participated in the study of the plexuses’ location, 12 were male and 7 were female. The patients' ages ranged from 26 to 92 years old, and the number of plexuses also varied, as shown in (Table 1): There was no correlation between the number of plexuses observed and the age or the post-mortem time (Fig. 5).

**Table 1:** Table demonstrating gender, age, time lapse between death and necropsy, number and approximate location of plexuses.

Donor	Gender	Age	*Time lapse*	Plexuses in 180 µm	Approximate location of the plexuses	Ganglion cells in 180 µm
1	Masc	87	12h	7	6µm ‒ 27µm ‒ 60µm ‒ 81µm ‒ 138µm ‒ 165µm ‒ 180µm	11
2	Fem	55	10h	10	6µm ‒ 18µm ‒ 24µm ‒ 63µm ‒ 78µm ‒ 102µm ‒ 114µm ‒ 120µm ‒ 138µm ‒ 174µm	13
3	Masc	43	10h	15	6µm ‒ 21µm ‒ 33µm ‒ 42µm ‒ 51µm ‒ 60µm ‒ 75µm ‒ 84µm ‒ 93µm ‒ 102µm ‒ 129µm ‒ 147µm ‒ 156µm ‒ 162µm ‒ 180µm	32
4	Masc	55	12h	12	12µm ‒ 18µm ‒ 45µm ‒ 54µm ‒ 87µm ‒ 114µm ‒ 120µm ‒ 135µm ‒ 141µm ‒ 153µm ‒ 171µm ‒ 177µm	15
5	Masc	26	10h	11	21µm ‒ 42µm ‒ 69µm ‒ 78µm ‒ 93µm ‒ 102µm ‒ 111µm ‒ 123µm ‒ 138µm ‒ 162µm ‒ 177µm	18
6	Fem	60	9h	8	30µm ‒ 57µm ‒ 69µm ‒ 78µm ‒ 93µm ‒ 111µm ‒ 135µm ‒ 165µm	12
7	Masc	71	10h	12	9µm ‒ 27µm ‒ 48µm ‒ 60µm ‒ 90µm ‒ 99µm ‒ 108µm ‒ 114µm ‒ 132µm ‒ 144µm ‒ 153µm ‒ 177µm	18
8	Fem	86	9h	9	12µm ‒ 27µm ‒ 51µm ‒ 78µm ‒ 141µm ‒ 153µm ‒ 165µm ‒ 174µm ‒ 180µm	12
9	Fem	92	10h	8	12µm ‒ 42µm ‒ 60µm ‒ 81µm ‒ 96µm ‒ 129µm ‒ 150µm ‒ 177µm	10
10	Masc	65	10h	14	9µm ‒ 24µm ‒ 30µm ‒48µm ‒ 69µm ‒ 84µm ‒ 96µm ‒ 105µm ‒ 114µm ‒ 129µm ‒ 141µm ‒ 160µm ‒ 169µm ‒ 180µm	28
11	Masc	65	7h	6	18µm ‒ 45µm ‒ 51µm ‒ 60µm ‒ 168µm ‒ 180µm	21
12	Masc	54	11h	10	12µm ‒ 27µm ‒ 36µm ‒ 54µm ‒ 87µm ‒ 105µm ‒ 120µm ‒ 141µm ‒ 150µm ‒ 180µm	19
13	Masc	47	11h	12	12µm ‒ 27µm ‒ 39µm ‒ 57µm ‒ 75µm ‒ 93µm ‒ 114µm ‒ 126µm ‒ 141µm ‒ 153µm ‒ 168µm ‒ 180µm	21
14	Masc	58	7h	12	3µm ‒ 24µm ‒ 36µm ‒ 57µm ‒ 69µm ‒ 87µm ‒ 99µm ‒ 114µm ‒ 132µm ‒ 153µm ‒ 165µm ‒ 180µm	22
15	Fem	83	12h	10	6µm ‒ 24µm ‒ 45µm ‒ 57µm ‒ 72µm ‒ 87µm ‒ 108µm ‒ 129µm ‒ 144µm ‒ 165µm	24
16	Masc	76	12h	10	3µm ‒ 21µm ‒ 45µm ‒ 69µm ‒ 90µm ‒ 81µm ‒ 111µm ‒ 129µm ‒ 144µm ‒ 162µm	22
17	Fem	89	8h	9	21µm ‒ 57µm ‒ 75µm ‒ 99µm ‒ 120µm ‒ 147µm ‒ 156µm ‒ 168µm ‒ 180µm	18
18	Fem	84	7h	11	12µm ‒ 21µm ‒ 39µm ‒ 66µm ‒ 84µm ‒ 99µm ‒ 114µm ‒ 126µm ‒ 144µm ‒ 162µm ‒ 177µm	22
19	Masc	52	11h	11	12µm ‒ 24µm ‒ 42µm ‒ 63µm ‒ 78µm ‒ 102µm ‒ 114µm ‒ 141µm ‒ 153µm ‒ 165µm ‒ 177µm	20

**Fig. 6:**
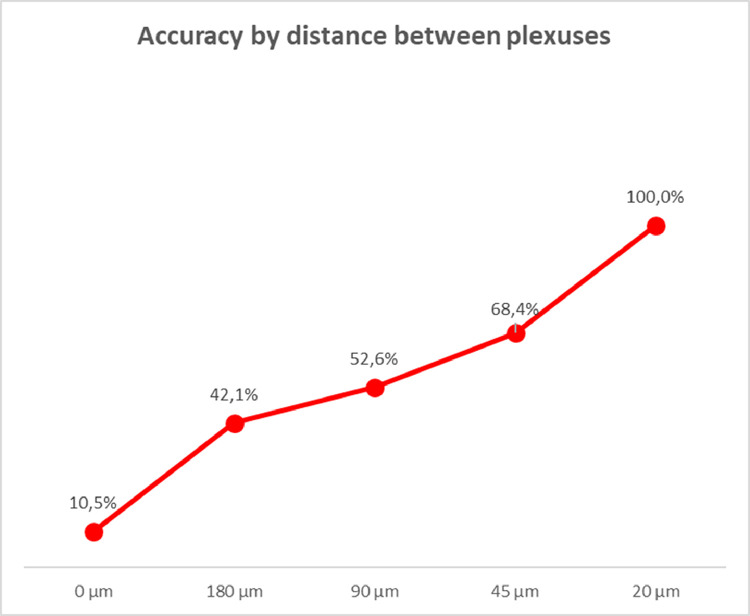
Graphic demonstrating the accuracy of reading by equidistant cuts.

The data obtained from the sample reading allowed us to analyze the diagnostic accuracy according to the distance between the plexuses. We verified that at every 20 µm of distance, it is possible to locate a ganglionic plexus in the submucosal area, with an accuracy of 100% (Fig. 6).

### Applying the systematization method to the reading of H&E-stained specimens

Out of the 47 specimens, 31 were collected from children with HD, and the other 16 were obtained from children who did not have the disease based on AChE screening. The reading method was applied to the analysis of these 47 biopsies from suspected HD patients, prepared using H&E staining. For this purpose, we performed an anatomopathological evaluation of every 20 µm of each fragment and compared our results with the diagnosis made by AChE. The accuracy obtained was 93.5%, with only three discordant cases. Validation values are shown in Table 2.

**Table 2:** Values for validation of the Hematoxylin-Eosin method.

**Accuracy**	0.935
**Sensitivity**	0.911
**Specificity**	1.000
**NPV**	0.812
**PPV**	1.000

## Discussion

In the current investigation, we made a refinement studying normal rectum fragments collected from adult cadavers, since it is easier to get samples from them when compared to getting samples from children. It is known that intestinal ganglion maturation is based on the maturation of ganglion cells, which may occur by the age of three weeks, so that was the reason why we collected rectal fragments from adult cadavers. Finally, in the present study, we were only interested in verifying whether ganglion cells were present in the adult normal rectum.^25^, ^26^, ^27^ Also, the authors may not consider the differences in ganglion cell counts between adults and children, as this number is constant over time, thanks to a mechanism of balance between apoptosis and neurogenesis.^26^

The only excluding factor that had to be considered was the time lapse between death and the rectum fragment collection, as in periods above twelve hours, tissue autolysis could occur. In addition, cadavers with any suspicion of Chagas disease were dismissed, as Chagas disease may cause acquired megacolon due to the inflammatory loss of myenteric ganglion cells.^23^

To confirm HD diagnosis, the immunohistochemical labeling of the calretinin method was chosen, because calretinin can specifically identify all ganglion cells in the histological cut. No ganglion cell went unnoticed by the observer during the reading of the slices.^15^^,^^28^ In addition, we observed a great variation in the number of neuronal cells and plexuses, between 10 and 32 cells, and between 7 and 15 plexuses per sample. Despite these variations, we could not show any correlation between the number of observed plexuses in the cadavers, which differs from previous evidence that with aging the intestinal plexuses could lose their cells.^26^^,^^29^ Also, we did not observe any correlation between the time elapsed after death in the moment of rectum sample collection and the number of cells observed in the analysis.

Regarding the analysis in equidistant histological cuts, the goal was to verify the distance between the ganglion plexuses. We started by analyzing the extremities (cut 1 and cut 60) and, after that, we inserted new cuts, always in the middle point between the previous two cuts, until we reached satisfactory accuracy. We found out that nine cuts 20 µm away from one another was a sufficient distance. Then, we applied this method to suspected HD cases, with an accuracy of 93.5%, similar to the specific methods utilized for HD diagnosis.^13^^,^^14^^,^^18^^,^^28^

When we compared the method of equidistant cuts and the AChE method for HD diagnosis, there were only three divergent cases, confirmed to be false positives, i.e., in nine cuts, no ganglion cells were found, although the patient did not show any HD clinical evidence.

Therefore, with this study, it is possible to affirm that by analyzing a rectal biopsy material every 20 µm there is a greater chance of visualizing a submucosal plexus in patients with normal bowel innervation.

It is important to point out as a limitation of this study the difficulty in obtaining samples from donors. In our initial project, we intended to collect and analyze at least 60 samples, but, due to the pandemic, SVOC-SP suspended all studies involving the collection of material between 2020‒2022, so we could collect only 19 samples. It is also worth mentioning that the process of preparing the slides and reading the samples from donors was very wearisome.

As a strength, we managed to achieve the result, by revisiting a technique as consolidated as H&E staining, so it was possible to create a more efficient method for HD diagnosis.

## Conclusion

In conclusion, the analysis of rectal submucosa every 20 µm showed there is a high probability of finding a ganglion plexus in bowels with normal innervation. This knowledge supported the formulation of a new, simpler, and faster method for pathological evaluation, to be utilized by pathologists for HD diagnosis.

## Availability of data and materials

The data supporting the conclusions are included in the article. Raw data are available upon request.

## Ethical approval and consent to participate

The ethics committees of Hospital das Clínicas ‒ University of Sao Paulo Medical School, Sao Paulo, Brazil approved this study (CAAE: 05219018.3.0000.0065). Consent to participate was required only in the first part of this study.

## Consent for publication

Not applicable

## Authors' contributions

SS conceived the research idea and designed this study with CL, MMS and ACAT. SS and JOG performed the histological techniques. SS and MMS analyzed the slides. SS, MMS and ACAT drafted the manuscript. UT revised the manuscript and finalized it. All authors read and approved the final manuscript.

## Funding

FAPESP Project no 2019/10339-1.

## Conflicts of interest

The authors declare no conflicts of interest.
